# Does antiretroviral therapy cause congenital malformations? A systematic review and meta-analysis

**DOI:** 10.4178/epih.e2021008

**Published:** 2021-02-03

**Authors:** Fekadu Mazengia Alemu, Alemayehu Worku Yalew

**Affiliations:** School of Public Health, College of Health Sciences, Addis Ababa University, Addis Ababa, Ethiopia

**Keywords:** Antiretroviral therapy, Congenital anomalies, Protease inhibitors, Integrase inhibitors, Efavirenz

## Abstract

**OBJECTIVES:**

This meta-analysis investigated the risk of congenital anomalies among infants of human immunodeficiency virus-infected pregnant women who were exposed to antiretroviral therapy (ART).

**METHODS:**

Cohort studies, case-control studies, randomized controlled trials, and controlled clinical trials were reviewed by searching MEDLINE/PubMed, Embase, Web of Science, Scopus, AIDSLINE, CINAHL, Cochrane Library, and Google/Google Scholar. Methodological quality was assessed using the GRADE evaluation. A DerSimonian and Laird random-effects model was used. Subgroup analyses and meta-regression were used to investigate heterogeneity.

**RESULTS:**

The electronic searches yielded 765 items. After quality assessment and grading, 30 studies were suitable for metaanalysis. In total, 1,461 congenital anomalies were found among 53,186 births. Children born to women receiving combined antiretroviral therapy (cART) had an approximately 10% higher risk of developing congenital anomalies (relative risk [RR], 1.09; 95% confidence interval [CI], 1.04 to 1.14). A subgroup analysis found no significant difference in the risk of congenital anomalies between cART and efavirenz users. However, zidovudine and protease inhibitor (RR, 1.09; 95% CI, 1.00 to 1.19) users were found to have a 10% increased risk of congenital anomalies, and integrase inhibitor users had a 60% increase in risk (RR, 1.61; 95% CI, 1.60 to 2.43). The subgroup results should be interpreted cautiously because of the moderate heterogeneity (I^2^ =58%).

**CONCLUSIONS:**

The use of protease inhibitors, integrase inhibitors, zidovudine, and newer drugs should be carefully considered in pregnant women. Further studies are needed to address environmental, nutrition, and adherence factors related to ART. Establishing a congenital anomalies surveillance system is recommended.

## INTRODUCTION

Human immunodeficiency virus (HIV) is a major indirect cause of maternal and neonatal morbidity and mortality, particularly in sub-Saharan African countries [1]. Preventing mother-to-child transmission of HIV (PMTCT) has been one of the greatest successes in efforts to combat the HIV pandemic [2]. The transmission of HIV has been sharply reduced by the use of PMTCT approaches combined with strategic use of highly active antiretroviral therapy (HAART) [3-5]. The benefits of antiretroviral therapy (ART) use during pregnancy considerably outweigh the potential risks. However, immediate and long-term harm in mothers and children, including congenital anomalies, remains a serious issue of concern [3,6,7].

A congenital anomaly is defined as the occurrence of a major structural or chromosomal defect or any group of 2 or more minor defects occurring in a newborn baby after 20 weeks of gestational age [8]. In under-resourced countries, various risk factors such as malnutrition, anemia, multi-parity, teenage pregnancy, limited healthcare access, comorbidities such as malaria, and irregular use of ART may increase the risk of birth defects. However, the role of such interactions in the development of birth defects has not been well studied [9].

A cohort study revealed that anemia (84%), neutropenia (64%), and thrombocytosis (24%) were the most common disorders among children exposed to ART. Long-term follow-up also revealed various neurological, cardiac, and ophthalmological pathologies [10].

The most commonly reported birth defects were found in the genital and urinary system (30.6%), the cardiovascular system (27.4%), the musculoskeletal system (12.9%), and the digestive system (9.7%) [11]. In zidovudine (ZDV)-exposed but HIV-uninfected infants, transient anemia, long-term hematological anomalies (neutropenia, thrombopenia, and lymphopenia), and hyperlactatemia have been reported. Preclinical studies showed that ZDV had a carcinogenic effect [12]. Research in the literature has also documented the timing of initiation and type of ART regimen as factors associated with adverse pregnancy outcomes [13].

For safety reasons, it is essential to monitor pregnancy outcomes in resource-limited settings during the design and implementation of the World Health Organization (WHO) ART guidelines [14]. The time of the highest sensitivity to teratogenic exposure is organogenesis, 18-60 days after conception or 4-13 weeks after the beginning of the last menstrual period [8]. However, congenital defects due to teratogenic exposure can happen any time through the course of pregnancy [15].

New drugs are coming to the market and effective ART is available, but toxicity issues and harmful outcomes during pregnancy, including major congenital anomalies, preterm delivery, anemia, and low birth weight, are of increasing concern among clinicians and program managers. [7,13,16-18]. There is a counter-argument that ART does not have an effect on the formation of congenital anomalies [3-5]. The main aim of this review, therefore, was to investigate the effect of ART on congenital anomalies and to derive a summary estimate.

## MATERIALS AND METHODS

The study designs reviewed include interventional studies (randomized controlled trials and controlled clinical trials). As Chou et al. [19] recommended gathering evidence on harms from a broad range of sources including observational studies, notably when clinical trials are lacking; observational studies (cohort and case-control studies) were also included.

### Search strategy and keywords

First, the DARE database (http://www.library.ucsf.edu) was explored in an attempt to confirm whether systematic reviews, meta-analyses, or ongoing projects related to this topic existed. Studies were selected and compared following the guidelines outlined in the Trent handbook [20] and Cochran’s hand book [21]. Following the implementation of the search strategy, the titles of all appropriate abstracts and titles collected from electronic and hand-searches were entered into EndNote version 6 (Clarivate Analytics, Philadelphia, PA, USA) reference software.

The main sources for the review were electronic bibliographic databases. The MEDLINE/PubMed and Embase databases, covering most areas of healthcare and containing index journals published from around the world, were searched. Furthermore, Web of Science, Scopus, and CINAHL were also searched. AIDSLINE, which focuses on a specific area of health, was also examined. The Cochrane Collaboration, an electronic database for reports of controlled trials (“CENTRAL”), and search engines such as Google Scholar were searched specifically for gray literature.

The following search terms were used as keywords and/or Medical Subject Headings (MeSH) terms:

((((Antiretroviral[All Fields] AND (“therapy”[Subheading] OR “therapy”[All Fields] OR “therapeutics”[MeSH Terms] OR “therapeutics”[All Fields])) AND (“congenital abnormal-ities” [MeSH Terms] OR (“congenital”[All Fields] AND “ab-normalities” [All Fields]) OR “congenital abnormalities”[All Fields] OR (“congenital”[All Fields] AND “anomalies”[All Fields]) OR “congenital anomalies”[All Fields])) AND (“congenital abnormalities” [MeSH Terms] OR (“congenital”[All Fields] AND “abnormalities” [All Fields]) OR “congenital abnormalities”[All Fields])) AND (“congenital abnormalities”[MeSH Terms] OR (“congenital”[All Fields] AND “abnor-malities”[All Fields]) OR “congenital abnormalities”[All Fields] OR (“congenital”[All Fields] AND “malformations” [All Fields]) OR “congenital malformations”[All Fields])) AND (“congenital abnormalities”[MeSH Terms] OR (“congenital”[All Fields] AND “abnormalities”[All Fields]) OR “congenital abnormal-ities”[All Fields] OR (“birth”[All Fields] AND “defect”[All Fields]) OR “birth defect”[All Fields])

### Eligibility criteria

Only studies fulfilling our eligibility criteria, defined using the population, intervention, comparator, outcome (PICO) framework, were included [22]:

(1) Population: Pregnant women living with HIV and/or children who were exposed to HIV or infected during pregnancy. Women at any gestational age were included, as the recent literature has suggested that focusing on congenital abnormalities only in the first trimester is outdated [15]. All countries and settings were eligible for inclusion.
(2) Interventions: All combinations and doses of ARTs. Standard combination antiretroviral therapy (cART) consists of a combination of antiretroviral drugs to maximally suppress HIV and stop the progression of disease. To be included, pregnant women should have taken ART for at least 1 month before delivery. Studies were not included if they examined ARTs that were directly administered to neonates, infants, or children; were pre-clinical animal studies; analyzed prophylaxis-only doses; or did not specify whether women were on ART.
(3) Comparators: Studies comparing antiretroviral medications administered to HIV-positive mothers to (i) HIV-negative women, (ii) HIV-positive women not receiving treatment, and (III) HIV-positive women taking another type of ART medication (medication classes as per the WHO 1a-1f category) were included. The last group was considered for a subgroup analysis to determine the effects of individual drugs on the occurrence of congenital anomalies.
(4) Outcomes: The presence or absence of congenital anomalies must have been stated and unambiguous. The primary safety outcome was major congenital malformations (overall and by specific type), as defined by the International Classification of Diseases, 10th revision [22], which includes congenital anomalies/birth defects in chapter XVII. Congenital anomalies were operationalized for this study as anatomical or functional defects, including metabolic ailments, which were present at birth [23].
(5) Study designs: Experimental (randomized clinical trials [RCTs] and non-RCTs), quasi-experimental (controlled before and after and time series), and observational (cohort, case-control, and drug registry) studies were included in the analysis. However, cross-sectional studies and case reports were excluded.
(6) Other limitations: No limitations were imposed on publication status, study site, and the duration of the study. However, non-English studies and those with a very small sample size (< 50) used to detect differences were excluded.

### Quality appraisal of papers and risk of bias

A structured template adopted from the Newcastle-Ottawa scale was used to appraise each paper. To facilitate the improvement in the quality of reporting of observational studies, the STROBE (Strengthening the Reporting of Observational Studies in Epidemiology) statement was used [24]. The quality criteria included whether sampling procedure and sample size calculations were illustrated, the description of algorithms and results known for both HIV-infected and uninfected women, the process of measurement of the outcome and the degree of blinding of the investigators about mothers’ infection status; the degree of follow-up; and the strategies used to control for confounding.

We used the GRADE approach to assess and grade the confidence of evidence for each outcome in the involved studies. Eight criteria were used to either downgrade or upgrade each study. As a rule of thumb, GRADE starts with a baseline rating of high for RCTs and low for non-RCTs, including observational studies. The five criteria used to downgrade the research quality are presence of risk of bias, inconsistency, indirectness of evidence, imprecision or lack of reliability, and publication bias.

If a serious concern exists, the evidence is downgraded by 1 level, such as from high to moderate (-1). If a very serious concern exists, the evidence is downgraded by 2 levels, such as from high to low (-2). The other three criteria (criteria 6 to 8) used to upgrade the grade are a large magnitude of effect, a dose-response effect, and all reasonable confounding factors reducing the effect (where an effect is observed) or suggesting a false effect (when no effect is observed). We judged whether the evidence should be upgraded once (+1) or twice (+2) for criteria 6-8. We integrated downgrading and upgrading factors to obtain an overall quality of evidence, ranked as high, moderate, low, or very low as specified by the GRADE approach. Finally, information concerning the quality of evidence for all outcomes was concisely combined in the “summary of findings” [15] (Supplementary Material 1). Subsequently, data from each of the 30 included studies were abstracted and entered into a data abstraction tool that was developed in Microsoft Excel 2013 (Microsoft, Redmond, WA, USA).

### Statistical analysis

We noted that there was variability among the included studies due to clinical variation (differences in the characteristics of participants, such as age and nutritional status, exposure, measurement, and categories of ART), differences in outcomes, and methodological variability (in study design). Using the Der-SimonianLaird random-effects model, an overall estimate of effect was determined [25]. For each study, the relative risk (RR) as a weighted measure of association was computed by comparing exposed to unexposed women or by comparing various ART regimens. This analysis also focused on the evaluation of the levels of discrepancy, the extent of dispersion, and the causes of heterogeneity. The heterogeneity assessment included study design, publication status, study setting, and drug categories. Stata version 14 (StataCorp., College Station, TX, USA) was used to analyze the data.

### Assessment of the extent of inconsistency

We used the eyeball technique on the forest plots to examine the dispersion of observed RRs. Along with the forest plot, we computed Cochran’s Q at p< 0.10 and the I^2^ statistic with a 95% confidence interval (CI) to formally quantify the proportion of variance in observed RRs that reflected the true heterogeneity between studies rather than mere chance [26]. A heterogeneity value with p< 0.10 demonstrated the existence of heterogeneity [27]. According to the criteria established by Higgins & Green [28], we considered I^2^ < 25% as indicating low heterogeneity, 50-75% as indicating substantial heterogeneity, and > 75% as indicating significant heterogeneity.

### Assessment of the amount of dispersion

In addition to I^2^, we also reported the τ^2^ statistic to indicate the amount of dispersion of true effects, mainly in the subgroup analysis. We computed the 95% CI to estimate the range within which a hypothetical new true measure of association was expected to be found in 95% of cases [29].

### Investigating the causes of heterogeneity

Subgroup analyses were done to explore how drug intervention, clinical variability, and study design variability influenced the pooled estimate. Even though both clinical and study design diversity leads to statistical heterogeneity, previous literature indicates that clinical aspects may vary across studies to a greater extent than design factors. The subgroup analyses in this study, therefore, focused on assessing the effects of key characteristics of participants that may be associated with the main outcomes of interest. Thus, we conducted subgroup analyses based on, but not limited to, antiretroviral drug class, publication status, fetal outcome, study setting, and methodological quality. We conducted a meta-regression using the study average or proportion values of the main participants’ characteristics [29].

### Meta-bias assessment

We used a funnel plot to explore the potential of publication bias and small study effect. Additionally, the Egger test and Harbord test for funnel plot asymmetry were conducted [27]. A trim-and-fill analysis was also conducted to see whether 1 or more studies influenced the estimate.

### Ethics statement

No ethical approval was required as this is a systematic review of published reviews and it did not involve any human participants.

## RESULTS

**Figure 1.**
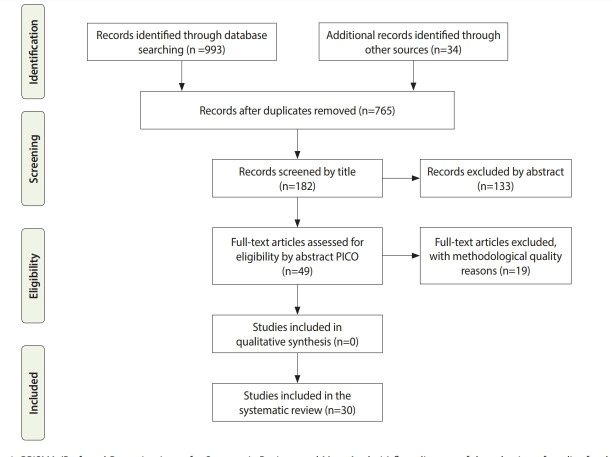
PRISMA (Preferred Reporting Items for Systematic Reviews and Meta-Analysis) flow diagram of the selection of studies for the systematic review and meta-analysis of associations of antiretroviral therapy with congenital anomalies. PICO, population, intervention, comparator, outcome.

The database searches produced 765 articles after duplicates were removed. The selection of titles and abstracts resulted in 182 potentially relevant articles, of which 133 references were excluded due to the reasons presented in Supplementary Material 2. Finally, 49 studies met the predefined inclusion criteria and PICO assessment. Thirty studies were suitable for a quantitative synthesis (meta-analysis). Most of the selected studies were designed as cohort studies. Figure 1 displays the flow of data through the different stages of the systematic review.

The study-specific estimates appeared considerably heterogeneous (e.g., the CIs of the following studies [3,7], did not overlap); hence, the fixed effect assumption might not be plausible for this dataset. This was reinforced by the Q-test, which also showed the presence of heterogeneity (p= 0.014). The mean of the I^2^ measure, which measures the amount of heterogeneity across studies, suggested the presence of moderate heterogeneity (39.8%). Therefore, we chose a random effect model for this particular study.

**Figure 2.**
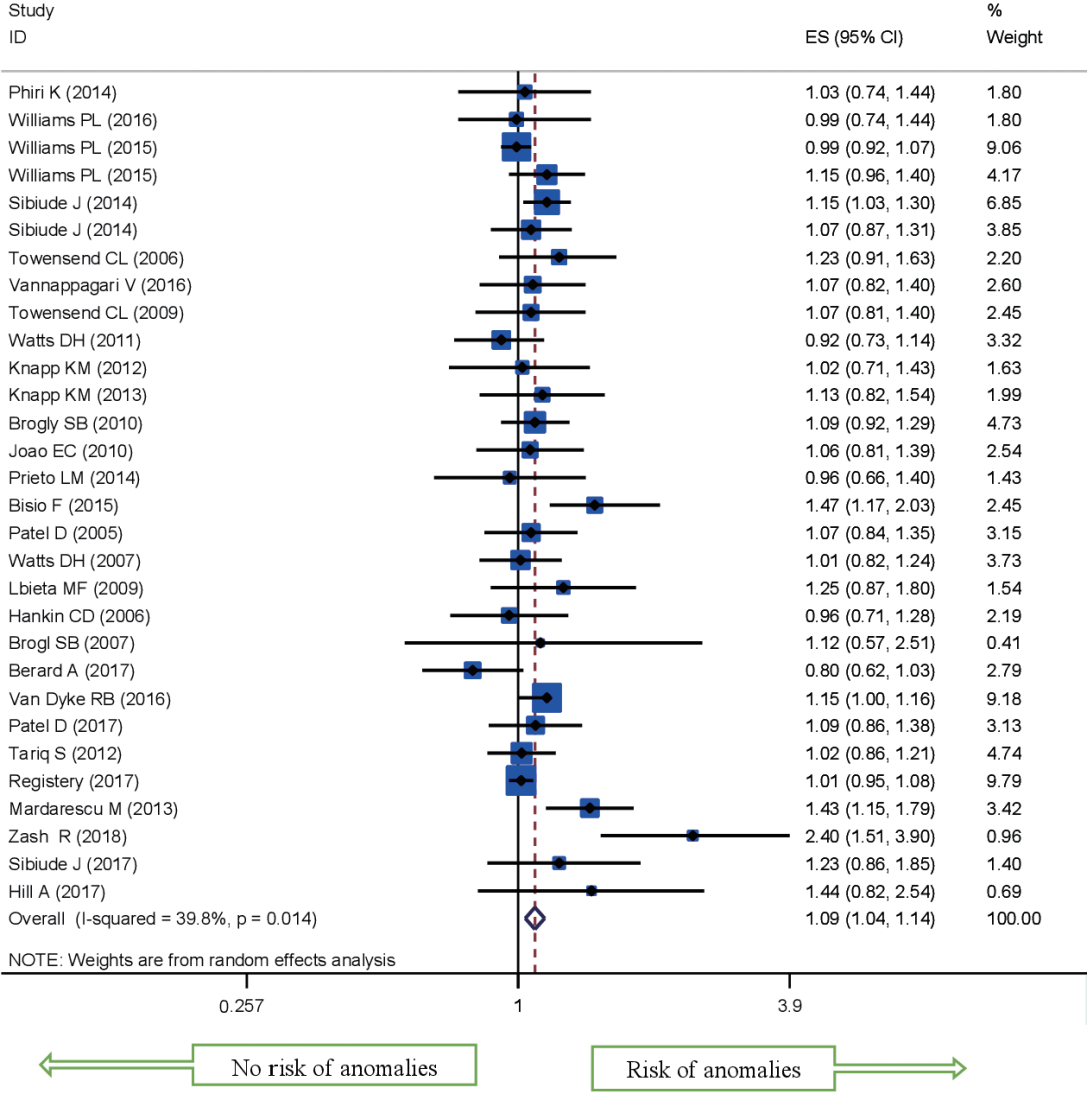
Meta-analysis of the effects of antiretroviral therapy (ART) on risk of congenital anomalies compared to ART-naïve individuals. ES, effect size; CI, confidence interval.

Of the 30 studies that were included in this review, 1,461 congenital anomalies were reported among a total of 53,186 births in women exposed to cART during pregnancy. For cART exposure during pregnancy, the pooled RR showed that those receiving cART had about a 10% increase in risk of having a child develop a congenital malformation compared to the non-exposed category (odds ratio [OR], 1.09; 95% CI, 1.04 to 1.14) (Figure 2).

### Heterogeneity assessment

#### Meta-regression

Several variables were entered into the model. Studies were compared according to their setting (developing or developed countries), study design (RCTs vs. observational studies), drug category (ZDV-based, nevirapine-based, efavirenz-based, protease inhibitor [PI]-based, or integrase inhibitor-based cART); methodological quality (high-grade [3+] and above vs. low-grade [1 to 2]), and publication status (published vs. unpublished). Some studies were done in developing countries, while others were done in developed counties. There were also differences in the ART regimen used among studies. Further, we suspected that study design, publication status, and methodological quality could moderate the differences observed in findings. As shown in Table 1, τ^2^ was 0.90, and I^2^ was 96.8%. The joint test for all 5 covariates gave a p-value of 0.006, indicating some evidence for an association of at least 1 of the covariates with the size of the treatment effect (Table 1).

#### Galbraith’s graph

**Figure 3.**
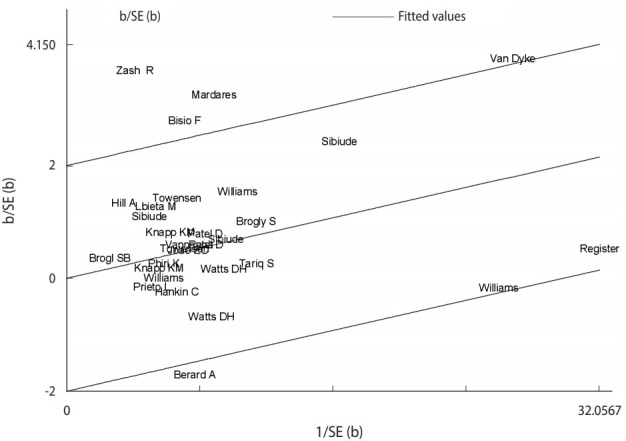
Galbraith plot for the log odds ratio of congenital anomalies in patients who received antiretroviral therapy (ART) versus ART-naïve individuals. b, beta; SE, standard error.

This graph represents the accuracy of each study versus the standardized effect. It also shows the adjusted regression line and sets 2 confidence bands. Five studies contributed to heterogeneity (Figure 3).

#### Subgroup analysis

**Figure 4.**
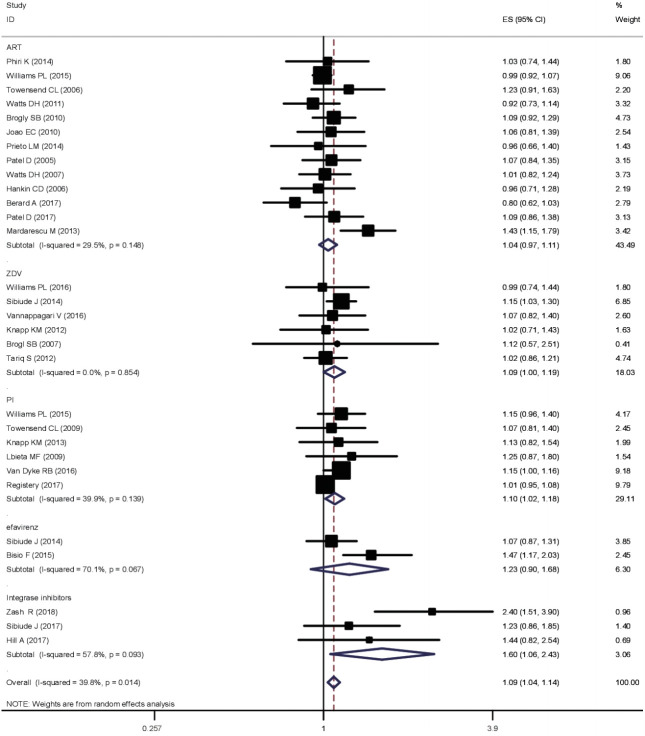
Subgroup analysis of the effects of different categories of ART on the risk of congenital anomalies. ES, effect size; CI, confiedence interval; ART, antiretroviral therapy; ZDV, zidovudine; PI, protease inhibitor.

The subgroup analysis found no differences in risk of congenital anomalies between pregnant women taking cART except PI based, ZDV based and integrase regimens to negative groups (RR, 1.04; 95% CI, 0.97 to 1.11). Heterogeneity between the studies was moderately low (I^2^ = 29%). Similarly, efavirenz exposure seemed not to have an association with congenital anomalies. However, a 10% increase in risk of congenital anomalies was shown for ZDV and PI-based antiretroviral therapies (RR, 1.09; 95% CI, 1.00 to 1.19 and RR, 1.10; 95% CI, 1.02 to 1.18, respectively) (Figure 4). Three studies reported congenital anomalies with a new group of drugs (integrase inhibitors) recently approved by the Food and Drug Administration and WHO. The subgroup analysis revealed a 60% increased risk of congenital anomalies associated with the use of integrase inhibitors (RR, 1.61; 95% CI, 1.60 to 2.43). However, the results for integrase inhibitors should be interpreted cautiously because of both the small sample size and the moderately high heterogeneity (I^2^ = 58%).

#### Summary of treatment effect

**Figure 5.**
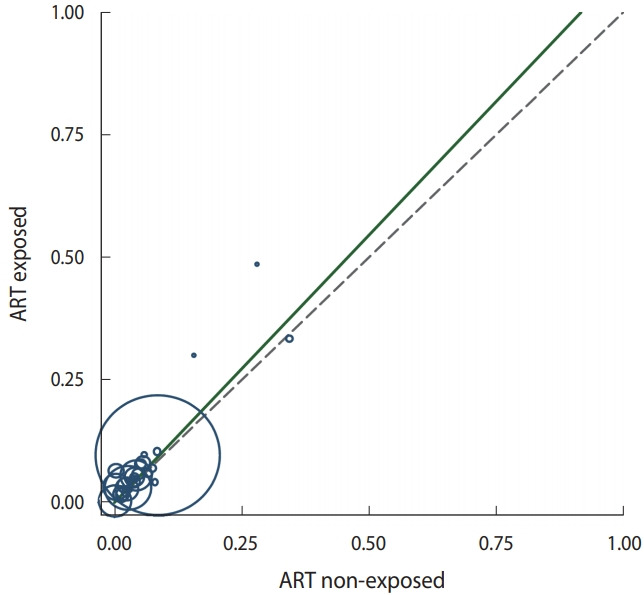
L’ Abbé plot depicting a summary of the effects of antiretroviral therapy (ART) on congenital malformation.

The L’Abbé plot (Figure 5) is another way to depict a summary of the effects of ART on congenital malformation. In the L’Abbé plot, studies are represented as follows: the risk of congenital anomalies (events) in the ART group (intervention group) is displayed on the y-axis and the event rate in the ART-naïve group (control group) on the x-axis. Each circle represents an individual study and the size of the circle is proportional to study size. The 45° line is the line of no effect. The summary effect shows that ART has a small but significant causal effect on congenital anomalies.

### Publication bias

#### Funnel plot and counter-funnel plot

**Figure 6.**
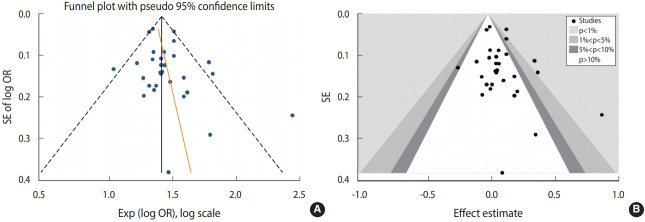
(A) meta-funnel and (B) counter-enhanced funnel with pseudo 95% confidence intervals to detect publication bias on the effect of antiretroviral therapy on congenital anomalies. SE, standard error; OR, odds ratio.

Funnel plots are scatter plots that display treatment effects from individual studies on the x-axis against a measure of study precision on the y-axis. Figure 6A plots the effect size against the variance of each study. Looking at the lower corner of the funnel plot, the slight emptiness of negative or null studies indicates a probability of publication bias [30]. The contour-enhanced funnel plot (Figure 6B) helps to differentiate between publication bias and other causes of observed asymmetry. Small studies were found not only in the shaded areas (statistical significance) but also in the white areas (areas of non-statistical significance). Thus, several factors, and not solely publication bias, might be responsible for the asymmetry observed in the funnel plot. The areas where missing studies were found included regions of both low and high statistical significance (i.e., the area crossed over the contours), suggesting that both studies showing ART and congenital anomalies to be non-significantly and significantly associated were missing. Therefore, publication bias cannot be established as the only cause of funnel asymmetry.

#### Regression analysis for publication bias

**Figure 7.**
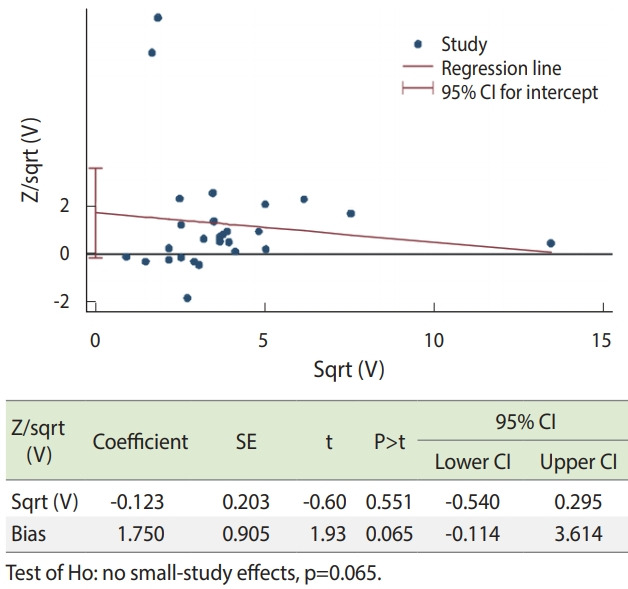
Harbord’s modified test and modified Gilbert’s plot for the small-study effect. Z, efficient score; V, scored variance; SE, standard error; CI, confidence interval.

Harbord’s meta-regression model was calculated to measure the scale and statistical significance of the association between observed effect sizes and the size of studies (Figure 7). Smaller studies did not tend to give different results when compared with larger trials, as the CI of the intercept contained the zero value. In addition, Harbord’s modified test for small-study effects showed that the estimated bias coefficient was 1.75 with a standard error of 0.905, giving a p-value of 0.065. The test provided weak evidence for the presence of small-study effects.

#### Trim and fill analysis

**Figure 8.**
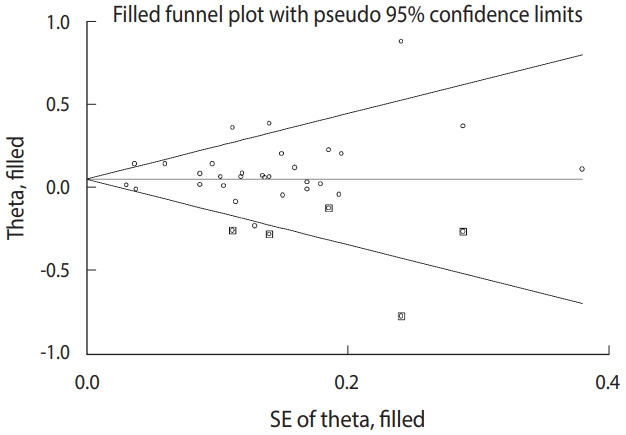
Trim- and- fill analysis of the small-study effect; 5 studies were trimmed and filled. SE, standard error.

A sensitivity analysis was performed to identify studies that had a larger influence on the estimates. Figure 8 provides a visual estimate with a 95% CI. The sensitivity analysis revealed that no individual studies affected the estimate excessively. The exclusion of reference [7] was influential, but the impact was not statistically significant. The trim-and-fill analysis revealed no significant asymmetry of the funnel plot (estimated effect size, 1.09; 95% CI, 1.14 to 3.56 vs. observed RR, 1.09; 95% CI, 1.04 to 1.14).

## DISCUSSION

This meta-analysis aimed to assess the extent to which exposure to ART during pregnancy is associated with congenital anomalies through a systematic review of published papers. This was assessed through measures of effect on the association and overall pooled estimates of these measures through a meta-analysis.

The pooled RR showed that mothers receiving cART were at about a 10% higher risk of having a child with a congenital malformation (RR, 1.09; 95% CI, 1.04 to 1.14) than the non-exposed category.

The connection between exposure to ART and the risk of adverse pregnancy outcomes, including congenital anomalies, has still not been clearly explained. Most studies were conducted in developed countries and were primarily limited by sample size, observational design, using patient registries or different comparison groups, and conflicting data [31]. In developing countries, for example, a study reported that micronutrient insufficiencies and exposure to environmental pollutants, as well as coinfections, may be more common in HIV-infected pregnant women, and this could result in increased prevalence of certain birth defects [16]. The other most likely hypothesis is that ART and immune reconstitution could modulate the Th1 to Th2 shift required by normal pregnancy [32]. Another proposed hypothesis is an etiological association between in utero Nucleoside/Nucleotide Reverse Transcriptase Inhibitors exposure and mitochondrial dysfunction [6,33-35]. Mitochondrial toxicity is associated with cognitive development and congenital anomalies [36].

The subgroup analysis yielded more specific results. Our findings support previous reports and meta-analyses stating that there is no association between efavirenz exposure and congenital anomalies [37]. However, a 10% increased risk of congenital anomalies was shown for ZDV and PI-based antiretroviral therapies (RR, 1.09; 95% CI, 1.00 to 1.19 and RR, 1.10; 95% CI, 1.02 to 1.18, respectively).

A large study in the United States revealed a similar finding. Although the pooled estimate showed that the absolute risk of congenital anomalies was relatively low, some individual drugs, such as atazanavir, showed relative increases in the risk of overall congenital anomalies and specific anomalies, which warrant further study [5,18]. Our cumulative meta-analysis also revealed there was no significant change over time, meaning the newest drugs may also cause congenital anomalies.

For instance, the proportion of neural tube defects among infants of women exposed to dolutegravir at conception is 3.16%. This percentage is higher than the proportion of 0.10% expected in the general population in sub-Saharan Africa, signifying that the result might not be explained by chance alone. The subgroup analysis revealed a 60% increased risk of congenital anomalies among integrase inhibitor users (RR, 1.61; 95% CI, 1.60 to 2.43). Therefore, integrase inhibitors should be warily studied.

One of the limitations of this review is the inclusion of literature written only in English. Furthermore, only a few studies conducted in developing countries were available, and some available ones were excluded at the methodological assessment stage due to the small number of enrolled participants.

## CONCLUSION

The findings of this review show that cART may not be a significant risk factor for congenital anomalies. It affirms the findings of previous systematic reviews that efavirenz does not have an association with congenital anomalies. In contrast, there was about a 10% increased risk of congenital anomaly deliveries among women who were exposed to PI-based regimens, ZDV, and integrase inhibitor treatments as compared to non-exposed individuals.

The number of HIV-exposed but uninfected infants who have been exposed to cART is rapidly increasing, especially in resourcelimited settings and as new drugs are introduced. There is an urgent need to carefully monitor the short-term and long-term consequences of antiretroviral drug exposure among pregnant women through surveillance systems. During the development of WHO or national ART guidelines, careful consideration should be taken in the inclusion of PI-based regimens, integrase inhibitors, ZDV, and newer drugs.

Further studies are needed in developing countries where environmental, nutrition, adherence, and other factors could influence the occurrence of congenital anomalies.

## Figures and Tables

**Table 1.** Meta-regression of the effects of covariates on the association of antiretroviral therapy and congenital anomalies^1^

Variables^2^	Coefficient	SD	t	P>t	95% CI	
					Lower CI	Upper CI
Setting	1.482	0.658	2.25	0.036	0.108	2.855
Study design	-0.037	0.306	-0.12	0.904	-0.675	0.601
Drug category	0.354	0.148	2.39	0.027	0.045	0.662
Methodological quality	-0.012	0.306	-0.04	0.970	-0.651	0.627
Publication status	0.761	0.496	1.53	0.141	-0.274	1.795
_cons	-1.905	1.138	-1.67	0.110	-4.280	0.470

SD, standard error; CI, confidence interval.

1 Between studies variation (τ^2^=0.005); Prob>F=0.006 (adjusted R^2^=43.40%).

2 Setting: compares studies from developed versus developing countries; Study design: compares randomized controlled trials with observational studies; Drug category: compares the six World Health Organization drug categories (1a-1f); Methodological quality: assesses the overall grading of strong versus weak; Publication status: compares published versus unpublished studies.

## Supplementary Materials

Supplementary Material 1. Methodological quality appraisal and Grading of Studies included in the systematic review

Supplementary Material 2. Excluded studies with reasons
