# Supplementary material for: Does antiretroviral therapy cause congenital malformations? A systematic review and meta-analysis
Source: Epidemiol Health. 2021 Feb 3;43:e2021008. doi: 10.4178/epih.e2021008 (PMC8060528; doi:10.4178/epih.e2021008)
Supplement: Supplementary Material 1. — Methodological quality appraisal and Grading of Studies included in the systematic review [file epih-43-e2021008-suppl1.docx]

Annex I. Methodological quality appraisal and Grading of Studies included in the systematic review

| First author: | Setting | Design | Quality level of a body of evidence _GRADE | | | | |  | NEWCASTLE - OTTAWA QUALITY ASSESSMENT SCALE | | |
| --- | --- | --- | --- | --- | --- | --- | --- | --- | --- | --- | --- |
|  |  |  | Limitations in the design & implementation | Indirectness Of Evidence | Unexplained heterogeneity | Imprecision of results | High probability of publication bias: | Grade | Selection | Comparability | Outcome |
| Phrill K  ([1](#_ENREF_1)) 2015 | USA | Tennessee Medicaid data linked to vital records | Yes | no | no | no | no | ++++strong | Yes based on mother claims ** | Good*** | used a 3-stage process  *** 8//9 |
| Vannappagari, V. 2016([2](#_ENREF_2)) | USA | Follow Up Data Registry | Yes  Exposure classification | yes | no | Yes | Yes | ++Low | *** somewhat representative*  secure record* | Can’t be ruled out effect of other intervention= 0 | ***  Registry & diagnostic base  Total =6 |
| Williams et.al.2016 ([3](#_ENREF_3)) | USA | Cohort  PHACS SMARTT study | no | YES | yes | No | no | +++  Strong | 2 drugs were used as HAART ** | Potential confounders like BMI cant be ruled out ** | Good***7/9 |
| Townsend, CL2009([4](#_ENREF_4)) | UK and Ireland | longitudinal | No | no | no | No | no | ++++  Strong | ***  Some women were Included more than once | **  injecting drug use (  ethnic origin,  maternal age at delivery & clinical status | ** **  Registry & diagnostic base  9 |
| Sibiude, J, 2014([5](#_ENREF_5)) | French | Cohort | no | no | yes | no | yes | ++++  Strong | *** | ** | ***  8/9 |
| Watts DH, 2011([6](#_ENREF_6)) | US, Brazil, the Bahamas | Cohort | no | yes | no | no | yes | +++ Moderate | *Use of registry  * comparison group | preconception initiation of ART was not  distinguished from initiation after conception | *multiple techniques used  **Use of standard case definition 5/9 |
| Knapp, KM 2012. ([7](#_ENREF_7)) | US and Europe | Longitudinal | Yes, Control Group were not clearly stated | no | yes | no | no | +++ Moderate | *Adequate Case defn  *Drawn from same community  *ascertainment of exposure  only 41 infants  with efavirenz exposure are included in this analysis. | - comparability not well discussed | - computerized screening  - recorded on clinical case  report forms - panel of clinicians who were  blinded to the mother’s ARV exposure during pregnancy. Deﬁn  itive  classiﬁcation of a congenital anomaly was made by  clinician consensus, using the Metropolitan Atlanta Congenital  Defects Program guidelines.  Total = 7/9 |
| Bera , 2010([8](#_ENREF_8)) | South Africa | Cohort | Yes, Classification bias | no | Yes | no | no | +++ Moderate | *There were switching to NVP after grouping | **pregnant woman were still on other HAART | *** U/S and Visual methods were used to ascertain  6/9 |
| Brogly, 2010([9](#_ENREF_9)) | USA | Cohort | No | no | no | no | Yes | ++++strong | ** from pro PACTG protocols 219 and 219C data | *** | ***Data measured every 3 months 8/9 |
| Zash R, 2016 ([10](#_ENREF_10)) | Botswana | Cohort | Yes | no | Yes  (nutrition status) | no | no | +++ Moderate | ***Adequate Case definition | *Comparison group are on ZDV | * Adverse pregnancy outcome instead of CA only |
| Joao, E. C. 2010([11](#_ENREF_11)) | Argentina  & Brazil | Cohort | No | no | Yes  (ART Grouping) | Yes | Yes | ++  Week | *Adequate Case defn  *Drawn from same community  *ascertainment of exposure | *Classification bias | **Use of Computer +Ultrasound  Use of standard classification  5/9 |
| Berard, A.  2017([12](#_ENREF_12)) | Canada | Cohort ( Population based) | Yes  *   inappropriate comparison group | No | no | yes | no | +++  Moderate | **  population-based and collected many potential confounders. | *   inappropriate comparison group  (significant imbalances in most background characteristics) | *  No mention of which congenital classification criteria |
| Delicio, Adriane M. 2018  ([13](#_ENREF_13)) | Brazil | Cohort (Retrospective) | Yes  *   inappropriate comparison group | Yes | Yes Children 0-5 years, Age difference | no | no | +++  Moderate | ** Large study  Retrospective | ** inappropriate comparison group | ** fair 6/9 |
| Van Dyke Dyke, 2016 ([14](#_ENREF_14)) | US, including Puerto Rico | Cohort | Not mentioned | Ye  S  The Objective was to study all outcomes | no | no | no | +++  Moderate | **Not mentioned however large sample size and sufficient follow-up was used | ** | **6/9 |
| Antiretroviral Pregnancy Registry Committee 2017([15](#_ENREF_15)) | USA | Longitudinal | Yes  *  Passive report   inappropriate comparison group | no | Yes | Yes based on clinician reports | no | ++ week | Large data  **  Selection bias | *no standard comparison. Usually made with population data | ** based on clinician reports  5/9 |
| Mărdărescu M. 2013 ([16](#_ENREF_16)) | Romania | Cohort | Yes | no | Yes  Duration of diseases, BMI | no | no | +++Moderate | ** Smaller sample size, selection bias | **fair | *fair 6/9 |
| Zash, R 2019 ([17](#_ENREF_17)) | Botswana | Cohort | Yes  Follow up study | Yes  Other ARTs not placebo | Yes  Only Midwives so Clinical Exam | no | no | ++  Low | *** No matching | ** No matching | - Only Midwives do the clinical   6/9 |
| Williams 2015 ([18](#_ENREF_18)) | USA | Cohort Prospective | No | No | Yes | no | No | ++++  Strong | **** | ** | **Outcome was based on document 8/9 |
| Townsend, CL2006  ([19](#_ENREF_19)) | UK and Ireland | longitudinal | No | no | no | No | no | ++++  Strong | ***  Some women were Included more than once | **  injecting drug use (  ethnic origin,  maternal age at delivery & clinical status | ** *  Registry & diagnostic base  7/9 |
| Prieto LM 2014([20](#_ENREF_20)) | Spain | Cohort | No | No | Yes. Use of Opiates | No | no | ++++  Strong | *** Women were included at any time | ** | *** European method of classification was used  8/9 |
| Bisio F, 2015 ([21](#_ENREF_21)) | Congo | Retrospective Cohort | Yes  Exposure classification | No | Yes. Other ARTs are also used | Yes | No | +++  Low | ** Women with NVP and other ART also includes | * | **No clear method of classification was used  5/9 |
| Patel D 2005 ([22](#_ENREF_22)) | Europe | Cohort | Yes ascertainment and reporting bias | No | No | No | No | ++++  Strong | *** ascertainment and reporting bias | ** | ** Reporting Bias  7/9 |
| Fernandez Ibieta M 2009([23](#_ENREF_23)) | Spain | Cohort | Yes  Analysis was  X2 or the Fisher test | Yes  It compares all ARTs | No | No | Yes  Wide CI and smaller comparison | ++ Week | ** Women with other ART also includes | - No clear comparison group | **No clear method of classification was used  5/9 |
| Hankin CD 2006 ([24](#_ENREF_24)) | UK | Cohort | Yes  Document review | No | No | No | No | ++++  Strong | *** ascertainment and reporting bias | ** | ** Reporting Bias  7/9 |
| Brogly SB  2007  ([25](#_ENREF_25)) | USA | Cohort | Yes | No | Yes  confounding by maternal viral load and psychoactive drug use | No | No | +++  Moderate | **  Exposure ascertainment  Controlling Confounding | ** | *** 7/9 |
| Tariq S etal. 2012([26](#_ENREF_26)) | Europe(European Collaborative study) | Cohort | Yes*** HAART for only at least 14 days rather than a month & Presence of other ARTs | Yes | No | No | No | +++Moderate | *** HAART for only at least 14 days rather than a month | *other HAARTs also include | ** Data for significant 192 participants were missing 6/9 |
| Hill A etal. ([27](#_ENREF_27)) | 6 studies | Systematic review | Mix up of studies  And only 6 studies included | No | Yes | Yes | Yes some are studies by pharmacological companies | ++ week | **  Compared at different exposure status.  Only two data bases searched | ** observational studies with out comparison | - Different outcome ascertainment 5/9 |
| Sibiude, J, etal. 2017([28](#_ENREF_28)) | French | Cohort | No | No | Yes other HAARTs | No | No | ++++  Strong | **** | *Yes other HAARTs | **Infected and uninfected children follow-up time  7/9 |

****Newcastle-Ottawa Assessment scale;*** *a study can be awarded a maximum number of stars within the selection, comparability and outcome categories. A maximum of 4 stars can be awarded for selection, 2 stars for comparability, and 3stars for outcome, a total score of 9 stars. We qualified studies with scores >5 to be methodologically fit****.***

***+ Grading was done according to the international GRADE group suggestion;****the system classifies quality of evidence (as reflected in confidence in estimates of effects) as high (Grade A ++++), moderate (Grade B++++), or low (Grade C++) according to factors that include the risk of bias, precision of estimates, the consistency of the results, and the directness of the evidence.*

1. Phiri K, Hernandez-Diaz S, Dugan KB, Williams PL, Dudley JA, Jules A, et al. First trimester exposure to antiretroviral therapy and risk of birth defects. The Pediatric infectious disease journal. 2014 Jul;33(7):741-6. PubMed PMID: 24445829. Pubmed Central PMCID: Pmc4420801. Epub 2014/01/22. eng.

2. Vannappagari V, Albano JD, Koram N, Tilson H, Scheuerle AE, Napier MD. Prenatal exposure to zidovudine and risk for ventricular septal defects and congenital heart defects: data from the Antiretroviral Pregnancy Registry. European journal of obstetrics, gynecology, and reproductive biology. 2016 Feb;197:6-10. PubMed PMID: 26687320. Epub 2015/12/22. eng.

3. Williams PL, Hazra R, Van Dyke RB, Yildirim C, Crain MJ, Seage GR, et al. Antiretroviral Exposure During Pregnancy and Adverse Outcomes in HIV-exposed Uninfected Infants and Children Using a Trigger-based Design: The SMARTT Study. AIDS (London, England). 2016;30(1):133-44. PubMed PMID: PMC4704129.

4. Townsend CL, Willey BA, Cortina-Borja M, Peckham CS, Tookey PA. Antiretroviral therapy and congenital abnormalities in infants born to HIV-infected women in the UK and Ireland, 1990-2007. AIDS (London, England). 2009 Feb 20;23(4):519-24. PubMed PMID: 19165088. Epub 2009/01/24. eng.

5. Sibiude J, Mandelbrot L, Blanche S, Le Chenadec J, Boullag-Bonnet N, Faye A, et al. Association between prenatal exposure to antiretroviral therapy and birth defects: an analysis of the French perinatal cohort study (ANRS CO1/CO11). PLoS medicine. 2014 Apr;11(4):e1001635. PubMed PMID: 24781315. Pubmed Central PMCID: Pmc4004551. Epub 2014/05/02. eng.

6. Watts DH, Li D, Handelsman E, Tilson H, Paul M, Foca M, et al. Assessment of birth defects according to maternal therapy among infants in the Women and Infants Transmission Study. Journal of acquired immune deficiency syndromes (1999). 2007 Mar 01;44(3):299-305. PubMed PMID: 17159659. Epub 2006/12/13. eng.

7. Katherine M. Knapp, Susan B. Brogly, Daniel G. Muenz, Hans M. L. Spiegel, Daniel H. Conway, Gwendolyn B. Scott, et al. Prevalence of Congenital Anomalies in Infants With In Utero Exposure to Antiretrovirals. The Pediatric infectious disease journal. 2012;31(2):164–70.

8. Bera E, McCausland K, Nonkwelo R, Mgudlwa B, Chacko S, Majeke B. Birth defects following exposure to efavirenz-based antiretroviral therapy during pregnancy: a study at a regional South African hospital. AIDS (London, England). 2010;24(2):283-9. PubMed PMID: 00002030-201001030-00014.

9. Brogly SB, Abzug MJ, Watts DH, Cunningham CK, Williams PL, Oleske J, et al. Birth defects among children born to human immunodeficiency virus-infected women: pediatric AIDS clinical trials protocols 219 and 219C. The Pediatric infectious disease journal. 2010 Aug;29(8):721-7. PubMed PMID: 20539252. Pubmed Central PMCID: Pmc2948952. Epub 2010/06/12. eng.

10. Zash R, Souda S, Chen JY, Binda K, Dryden-Peterson S, Lockman S, et al. Reassuring Birth Outcomes With Tenofovir/Emtricitabine/Efavirenz Used for Prevention of Mother-to-Child Transmission of HIV in Botswana. Journal of acquired immune deficiency syndromes (1999). 2016 Apr 01;71(4):428-36. PubMed PMID: 26379069. Pubmed Central PMCID: Pmc4767604. Epub 2015/11/01. eng.

11. Joao EC, Calvet GA, Krauss MR, Freimanis Hance L, Ortiz J, Ivalo SA, et al. Maternal antiretroviral use during pregnancy and infant congenital anomalies: the NISDI perinatal study. Journal of acquired immune deficiency syndromes (1999). 2010 Feb;53(2):176-85. PubMed PMID: 20104119. Pubmed Central PMCID: Pmc2901917. Epub 2010/01/28. eng.

12. Caniglia EC, Zash R, Jacobson DL, Diseko M, Mayondi G, Lockman S, et al. Emulating a target trial of antiretroviral therapy regimens started before conception and risk of adverse birth outcomes. AIDS (London, England). 2018 Jan 2;32(1):113-20. PubMed PMID: 29112066. Pubmed Central PMCID: PMC5718935. Epub 2017/11/08. eng.

13. Naicker N, Naidoo A, Werner L, Garrett N, Majola N, Asari V, et al. Efficacy and safety of tenofovir-containing antiretroviral therapy in women who acquired HIV while enrolled in tenofovir gel prophylaxis trials. Antiviral therapy. 2017;22(4):287-93. PubMed PMID: 27835613. Epub 2016/11/12. eng.

14. Van Dyke RB, Chadwick EG, Hazra R, Williams PL, Seage GR, 3rd. The PHACS SMARTT Study: Assessment of the Safety of In Utero Exposure to Antiretroviral Drugs. Frontiers in immunology. 2016;7:199. PubMed PMID: 27242802. Pubmed Central PMCID: PMC4876360. Epub 2016/06/01. eng.

15. Committee APRS. Antiretroviral Pregnancy Registry Interim Report for 1 January 1989 through 31 July 2019. Wilmington, NC: Registry Coordinating Center, 2017.

16. Mărdărescu M, Petre C, Streinu-Cercel A, Petrea S, Neagu-Drăghicenoiu R, Ungurianu R, et al. Surveillance of mother to child transmission of HIV in Romania, a 12 years’ experience in the National Institute for Infectious Diseases “Prof. Dr. Matei Balş”. BMC infectious diseases. 2013 2013/12/16;13(1):O1.

17. Zash R, L H, Diseko M., Jacobson D., Brummel S., Mayondi G., et al. Neural tube defects by antiretroviral and HIV exposure in the Tsepamo Study, Botswana IAS 2019 Conference Mexico 2019.

18. Williams PL, Crain MJ, Yildirim C, Hazra R, Van Dyke RB, Rich K, et al. Congenital anomalies and in utero antiretroviral exposure in human immunodeficiency virus-exposed uninfected infants. JAMA pediatrics. 2015 Jan;169(1):48-55. PubMed PMID: 25383770. Pubmed Central PMCID: Pmc4286442. Epub 2014/11/11. eng.

19. Townsend CL, Tookey PA, Cortina-Borja M, Peckham CS. Antiretroviral therapy and congenital abnormalities in infants born to HIV-1-infected women in the United Kingdom and Ireland, 1990 to 2003. Journal of acquired immune deficiency syndromes (1999). 2006 May;42(1):91-4. PubMed PMID: 16763496. Epub 2006/06/10. eng.

20. Prieto LM, Gonzalez-Tome MI, Munoz E, Fernandez-Ibieta M, Soto B, Alvarez A, et al. Birth defects in a cohort of infants born to HIV-infected women in Spain, 2000-2009. BMC infectious diseases. 2014 Dec 24;14:700. PubMed PMID: 25808698. Pubmed Central PMCID: Pmc4297442. Epub 2014/01/01. eng.

21. Bisio F, Nicco E, Calzi A, Giacobbe DR, Mesini A, Banguissa H, et al. Pregnancy outcomes following exposure to efavirenz-based antiretroviral therapy in the Republic of Congo. The new microbiologica. 2015 Apr;38(2):185-92. PubMed PMID: 25938743. Epub 2015/05/06. eng.

22. Patel D, Thorne C, Fiore S, Newell ML. Does highly active antiretroviral therapy increase the risk of congenital abnormalities in HIV-infected women? Journal of acquired immune deficiency syndromes (1999). 2005 Sep 01;40(1):116-8. PubMed PMID: 16123696. Epub 2005/08/27. eng.

23. Fernandez Ibieta M, Ramos Amador JT, Bellon Cano JM, Gonzalez-Tome MI, Guillen Martin S, Navarro Gomez M, et al. [Birth defects in a cohort of uninfected children born to HIV-infected women]. Anales de pediatria (Barcelona, Spain : 2003). 2009 Mar;70(3):253-64. PubMed PMID: 19246263. Epub 2009/02/28. Malformaciones congenitas en una cohorte de ninos no infectados, hijos de madres infectadas por el virus de la inmunodeficiencia humana. spa.

24. Hankin CD. Exposure to antiretroviral therapy in uninfected children born to HIV infected women in Europe: University of London 2006.

25. Brogly SB, Jean-Philippe P, Ylitalo N. In utero nucleoside reverse transcriptase inhibitor exposure and signs of possible mitochondrial dysfunction in HIV-uninfected children. AIDS (London, England). 2007;21(8):929-38.

26. Tariq S, Townsend CL, Cortina-Borja M, Duong T, Elford J, Thorne C, et al. Use of zidovudine-sparing HAART in pregnant HIV-infected women in Europe: 2000-2009. Journal of acquired immune deficiency syndromes (1999). 2011;57(4):326-33. PubMed PMID: 21499113. eng.

27. Hill A, Clayden P, Thorne C, Christie R, Zash R. Safety and pharmacokinetics of dolutegravir in HIV-positive pregnant women: a systematic review. J Virus Erad. 2018;4(2):66-71. PubMed PMID: 29682297. eng.

28. J. Sibiude, J. Warszawski, S. Blanche, O. Dialla, A. Faye, C. Dollfus, et al. Evaluation of the risk of birth defects among children exposed to raltegravir in utero in the ANRS-French Perinatal Cohort EPF - Raltegravir Not Tied to Birth Defects in French Study of 479 Births IAS 2017: Conference on HIV Pathogenesis Treatment and Prevention; Paris, France 2017
