# Supplementary material for: Does antiretroviral therapy cause congenital malformations? A systematic review and meta-analysis
Source: Epidemiol Health. 2021 Feb 3;43:e2021008. doi: 10.4178/epih.e2021008 (PMC8060528; doi:10.4178/epih.e2021008)
Supplement: Supplementary Material 2. — Excluded studies with reasons [file epih-43-e2021008-suppl2.docx]

Annex II:

Supplementary Table 2: Excluded studies with reasons

| **Study ID** | **Country** | **Reason For Exclusion** |
| --- | --- | --- |
| Liu KC , 2014 ([1](#_ENREF_1)) | South Africa and Zambia | No Comparative group  Data from un exposed group is missing  have missed nonviable  anomalies  examinations by midwives was not validated for proficiency to detect  congenital anomalies, and so birth defects may be underestimated. |
| Ajibola G ([2](#_ENREF_2)) | Botswana | Indirectness of evidence from cotrimoxazole vs. placebo study  Week Statistical Analysis |
| Bussmann, Hermann, 2013  ([3](#_ENREF_3)) | Botswana | Very small sample size 22 people unable to detect the effect  problem on design |
| Mugo NR([4](#_ENREF_4)) | Kenya | Only look in to a single HAART |
| Assaye, 2011 ([5](#_ENREF_5)) | Ethiopia | Didn’t directly measure birth outcome  No comparison group  NP ART Group  Indirectness of the finding |
| Et.al 2011([6](#_ENREF_6)). | Nigeria | Only look in to single CA  Based on case series |
| Jeffrey S. A. Stringer, 2013 ([7](#_ENREF_7)) | Cameroon, Cote D'Ivoire, South Africa, and Zambia | Outcome is measured after 2 years  Indirectness of the finding |
| Liu, K. C. et.al 2014 ([1](#_ENREF_1)) | Zambia, South Africa | No comparison group,  Confounders were not controlled |
| White A,1997 ([8](#_ENREF_8)) | Norway | Indirectness of the finding  Non intervention group not well ascertained |
| Watts DH 2007([9](#_ENREF_9)) | USA | No comparison group |
| Watts DH 2011([10](#_ENREF_10)) | USA | Compares first trimester with 3^rd^ not among exposed not exposed |
| Thorne C, 2005([11](#_ENREF_11)) | Expert Opinion | Indirectness of evidence |
| Roberts SS ([12](#_ENREF_12)) | USA | Comparison group is different from exposed group (have different exposure status ) |
| Wang L, 2013([13](#_ENREF_13)) | Systematic review | Systematic Review |
| Ekouevi DK, 2011([14](#_ENREF_14)) | Cote d'Ivoire | Outcome on congenital anomalies was zero due to small sample size |
| Florida M 2013([15](#_ENREF_15)) | Italy | Comparison between transmission and Ethnicity than ART exposure |
| Hsu HE, 2011 ([16](#_ENREF_16)) | USA | Data is based on rate and data is reported based on projected Rate than actual finding |
| Nielsen-Saines K, 2012([17](#_ENREF_17)) | India, Thailand, Brazil | Small sample size (Small data in each group) |
|  |  |  |
| Cressey TR, 2012 ([18](#_ENREF_18)) | International (USA) | small Sample size (25), Outcome zero |
| Manosuthi W, 2004 ([19](#_ENREF_19)) | Thailand | small Sample size (29 VS 24 in the exposed and control group) |
| Jibril M , 2013([20](#_ENREF_20)) | Nigeria | Small Sample to detect meaningful outcome (only 2 cases) |
| Jungmann EM, 2001([21](#_ENREF_21)) | UK, London | Small Sample to detect meaningful outcome (only 9 cases)  Non specificity (ART +foliate antagonists) |
| Tudor AM , 2014 ([22](#_ENREF_22)) | Romania | No Control Group |
|  |  |  |

1. Liu KC, Farahani M, Mashamba T, Mawela M, Joseph J, Van Schaik N, et al. Pregnancy outcomes and birth defects from an antiretroviral drug safety study of women in South Africa and Zambia. AIDS (London, England). 2014 Sep 24;28(15):2259-68. PubMed PMID: 25115319. Epub 2014/08/15. eng.

2. Ajibola G, Zash G, Shapiro R, Batlang O, Botebele K, Bennett K, et al. Detecting congenital malformations - Lessons learned from the Mpepu study, Botswana. PloS one. 2017;12(3).

3. Bussmann H, Wester CW, Wester CN, Lekoko B, Okezie O, Thomas AM, et al. Pregnancy rates and birth outcomes among women on efavirenz-containing highly active antiretroviral therapy in Botswana. Journal of acquired immune deficiency syndromes (1999). 2007 Jul 01;45(3):269-73. PubMed PMID: 17450102. Epub 2007/04/24. eng.

4. Mugo NR, Hong T, Celum C, Donnell D, Bukusi EA, John-Stewart G, et al. Pregnancy incidence and outcomes among women receiving preexposure prophylaxis for HIV prevention: a randomized clinical trial. Jama. 2014 Jul 23-30;312(4):362-71. PubMed PMID: 25038355. Pubmed Central PMCID: Pmc4362516. Epub 2014/07/20. eng.

5. Chekol A. Birth outcome and HIV infection among labouring women in Assosa Hospital, Southwest Ethiopia. Ethiop J Health Dev. 2011;25(1):10-6.

6. James A, Oluwatosin B, Njideka G, Babafemi, Benjamin OG, Olufemi D, et al. CLEFT PALATE IN HIV-EXPOSED NEWBORNS OF MOTHERS ON HIGHLY ACTIVE ANTIRETROVIRAL THERAPY. Oral surgery. 2014 Dec;7(Suppl 1):102-6. PubMed PMID: 25653715. Pubmed Central PMCID: Pmc4313880. Epub 2015/02/06. eng.

7. Stringer JS, Stinson K, Tih PM, Giganti MJ, Ekouevi DK, Creek TL, et al. Measuring coverage in MNCH: population HIV-free survival among children under two years of age in four African countries. PLoS medicine. 2013;10(5):e1001424. PubMed PMID: 23667341. Pubmed Central PMCID: PMC3646218. Epub 2013/05/15. eng.

8. White A, Eldridge R, Andrews E. Birth outcomes following zidovudine exposure in pregnant women: the Antiretroviral Pregnancy Registry. Acta paediatrica (Oslo, Norway : 1992) Supplement. 1997 Jun;421:86-8. PubMed PMID: 9240865. Epub 1997/06/01. eng.

9. Watts DH, Li D, Handelsman E, Tilson H, Paul M, Foca M, et al. Assessment of birth defects according to maternal therapy among infants in the Women and Infants Transmission Study. Journal of acquired immune deficiency syndromes (1999). 2007 Mar 01;44(3):299-305. PubMed PMID: 17159659. Epub 2006/12/13. eng.

10. Watts DH, Huang S, Culnane M, Kaiser KA, Scheuerle A, Mofenson L, et al. Birth defects among a cohort of infants born to HIV-infected women on antiretroviral medication. Journal of perinatal medicine. 2011 Mar;39(2):163-70. PubMed PMID: 21142844. Pubmed Central PMCID: Pmc3068472. Epub 2010/12/15. eng.

11. Thorne C, Newell ML. The safety of antiretroviral drugs in pregnancy. Expert opinion on drug safety. 2005 Mar;4(2):323-35. PubMed PMID: 15794723. Epub 2005/03/30. eng.

12. Roberts SS, Martinez M, Covington DL, Rode RA, Pasley MV, Woodward WC. Lopinavir/ritonavir in pregnancy. Journal of acquired immune deficiency syndromes (1999). 2009 Aug 01;51(4):456-61. PubMed PMID: 19381099. Epub 2009/04/22. eng.

13. 96+Wang L, Kourtis AP, Ellington S, Legardy-Williams J, Bulterys M. Safety of tenofovir during pregnancy for the mother and fetus: a systematic review. Clinical infectious diseases : an official publication of the Infectious Diseases Society of America. 2013 Dec;57(12):1773-81. PubMed PMID: 24046310. Epub 2013/09/21. eng.

14. Ekouevi DK, Coffie PA, Ouattara E, Moh R, Amani-Bosse C, Messou E, et al. Pregnancy outcomes in women exposed to efavirenz and nevirapine: an appraisal of the IeDEA West Africa and ANRS Databases, Abidjan, Cote d'Ivoire. Journal of acquired immune deficiency syndromes (1999). 2011 Feb 01;56(2):183-7. PubMed PMID: 21084995. Pubmed Central PMCID: Pmc3045727. Epub 2010/11/19. eng.

15. Floridia M, Mastroiacovo P, Tamburrini E, Tibaldi C, Todros T, Crepaldi A, et al. Birth defects in a national cohort of pregnant women with HIV infection in Italy, 2001-2011. BJOG : an international journal of obstetrics and gynaecology. 2013 Nov;120(12):1466-75. PubMed PMID: 23721372. Epub 2013/06/01. eng.

16. Hsu HE, Rydzak CE, Cotich KL, Wang B, Sax PE, Losina E, et al. Quantifying the risks and benefits of efavirenz use in HIV-infected women of childbearing age in the USA. HIV medicine. 2011 Feb;12(2):97-108. PubMed PMID: 20561082. Pubmed Central PMCID: Pmc3010302. Epub 2010/06/22. eng.

17. Nielsen-Saines K, Komarow L, Cu-Uvin S, Jourdain G, Klingman KL, Shapiro DE, et al. Infant outcomes after maternal antiretroviral exposure in resource-limited settings. Pediatrics. 2012 Jun;129(6):e1525-32. PubMed PMID: 22585772. Pubmed Central PMCID: Pmc3362906. Epub 2012/05/16. eng.

18. Cressey TR, Stek A, Capparelli E, Bowonwatanuwong C, Prommas S, Sirivatanapa P, et al. Efavirenz Pharmacokinetics during the Third Trimester of Pregnancy and Postpartum. Journal of acquired immune deficiency syndromes (1999). 2012;59(3):245-52. PubMed PMID: PMC3288559.

19. Manosuthi W, Sungkanuparph S, Vibhagool A, Rattanasiri S, Thakkinstian A. Nevirapine- versus efavirenz-based highly active antiretroviral therapy regimens in antiretroviral-naïve patients with advanced HIV infection. HIV medicine. 2004;5(2):105-9.

20. Jibril M, Egunsola O. Is tenofovir/emtricitabine teratogenic? Therapeutic Advances in Drug Safety. 2013;4(3):115-7. PubMed PMID: PMC4110866.

21. Jungmann EM, Mercey D, DeRuiter A, Edwards S, Donoghue S, Booth T, et al. Is first trimester exposure to the combination of antiretroviral therapy and folate antagonists a risk factor for congenital abnormalities? Sexually transmitted infections. 2001 Dec;77(6):441-3. PubMed PMID: 11714944. Pubmed Central PMCID: Pmc1744398. Epub 2001/11/21. eng.

22. Tudor AM. Severe birth defects in children perinatal exposed to HIV from a "real-world" setting: Infectious Diseases National Institute, Bucharest, Romania. Journal of the International AIDS Society. 2014;17(4 Suppl 3):19699. PubMed PMID: 25397447. Pubmed Central PMCID: Pmc4225364. Epub 2014/11/15. eng.
